# Association between religiosity and happiness in patients with chronic kidney disease on hemodialysis

**DOI:** 10.1590/2175-8239-JBN-2018-0096

**Published:** 2018-11-08

**Authors:** Janaína Siqueira, Natália Maria Fernandes, Alexander Moreira-Almeida

**Affiliations:** 1 Universidade Federal de Juiz de Fora, Faculdade de Medicina, NUPES - Núcleo de Pesquisas em Espiritualidade e Saúde, Juiz de Fora, MG, Brasil.

**Keywords:** Renal Insufficiency, Chronic, Kidney Failure, Chronic, Renal Dialysis, Religion, Spirituality, Quality of Life, Insuficiência Renal Crônica, Falência Renal Crônica, Diálise Renal, Religião, Espiritualidade, Qualidade de Vida

## Abstract

**Objectives:**

Religiosity/spirituality (R/S) seems to be a relevant factor in chronic diseases adaptation, but there is a lack of studies involving chronic kidney disease (CKD). This study aimed to investigate the association between R/S and happiness among CKD patients on hemodialysis and whether Sense of Coherence (SC) mediates this possible association.

**Methods:**

This was a cross-sectional study in two renal replacement therapy centers in Brazil, involving 161 adults on hemodialysis. Linear regressions were performed to evaluate the association between R/S (predicting variable measured with Duke Religious Index - DUREL) and happiness (outcome variable), adjusted for sociodemographic, clinical, and some laboratory variables. Later, SC was added to the model to test the possible mediating effect.

**Results:**

Most patients (91.20%) reported some religious affiliation. Private Religiosity (PR) (β = 0.53; 95% CI = 0.01 a 1.06), Intrinsic Religiosity (IR) (β = 0.48; 95% CI = 0.18 a 0.79), and SC (β = 0.11; 95% CI = -0.09 a 0.15) correlated with higher levels of happiness, controlling for clinical and sociodemographic variables. When SC was included in the model, IR (β = 0.34; 95% CI = 0.07 to 0.60) and SC (β = 0.11; 95% CI = 0.08 to 0.14) remained significantly. No clinical or sociodemographic variable correlated with happiness.

**Conclusions:**

Patients on hemodialysis showed high levels of R/S, which correlated with higher happiness levels. Clinical and sociodemographic variables were not correlated with patients' happiness. Psychosocial variables such as R/S and SC are potential key targets for interventions to promote better survival quality among CKD patients.

## Resumo

**Objetivos:**

Religiosidade/espiritualidade (R/E) parecem ser fatores relevantes na adaptação a doenças crônicas, mas faltam estudos nessa área envolvendo pacientes com doença renal crônica (DRC). O presente estudo teve como objetivo investigar a associação entre R/E e felicidade em pacientes com DRC em hemodiálise e se o senso de coerência (SC) faz a mediação dessa possível associação.

**Métodos:**

Estudo transversal realizado em dois centros de terapia renal substitutiva no Brasil com 161 adultos em hemodiálise. Regressões lineares foram utilizadas para avaliar a associação entre R/E (variável preditora medida pelo Índice de Religiosidade de Duke - DUREL) e felicidade (variável de desfecho), ajustadas para variáveis sociodemográficas, clínicas e algumas variáveis laboratoriais. Posteriormente, o SC foi acrescentado ao modelo para testar seu possível efeito mediador.

**Resultados:**

A maioria dos pacientes (91,20%) relatou alguma afiliação religiosa. Religiosidade Privada (RP) (β = 0,53; IC 95% = 0,01 a 1,06) e Religiosidade Intrínseca (RI) (β = 0,48; IC 95% = 0,18 a 0,79) e SC (β = 0,11; IC 95% = -0,09 a 0,15) foram correlacionadas a níveis mais elevados de felicidade após controle para variáveis clínicas e sociodemográficas. Quando SC foi incluído no modelo, IR (β = 0.34; 95% IC = 0.07 a 0.60) and SC (β = 0.11; 95% IC = 0.08 a 0.14) continuaram se correlacionando significativamente com felicidade. Nenhuma variável clínica ou sociodemográfica apresentou correlação com felicidade.

**Conclusões:**

Os pacientes em hemodiálise apresentaram altos níveis de R/E, que por sua vez foi correlacionada com elevados níveis de felicidade. As variáveis clínicas e sociodemográficas não exibiram correlação com a felicidade dos pacientes. Variáveis psicossociais como R/E e SC são possíveis alvos para intervenções destinadas a promover a melhora da qualidade de sobrevida dos pacientes com DRC.

## Introduction

Chronic kidney disease (CKD) is a health problem of increasing importance. The overall prevalence of CKD in the general US population was 2,067 patients per million in 2014^1^ and the prevalence in Latin America was 660 patients per million in 2010^2^.

For category 5 of CKD, requiring renal replacement therapy, only a successful renal transplant can offer renal function close to normal. The treatment process is often a difficult experience^3^ and the most frequent psychological conditions found in these patients are anxiety, stress, and depression^4^. There is also a decrease in quality of life (QoL)^3^.

We adopted a concept proposed by Lyubomirsky, in which happiness is the sum of two parts: positive emotions and the perception that life has meaning^5^^,^ 6. Happy people tend to be more successful in various domains of life,^7^^,^ 8 but identifying the factors that generate happiness remains a challenge.

A growing number of studies report that higher levels of religious involvement tend to be associated with happiness, better well-being and physical health, lower rates of depression, suicide, substance use/abuse and overall mortality,^9^^,^ 10 and improved adaptation to chronic diseases^11^. Religiosity/Spirituality (R/S) was also considered a relevant factor of QoL in patients with CKD^12^^,^ 13 ^,^ 14.

Spirituality is the relationship with the sacred and the transcendent, which may or may not lead to the development of a religion. Religiosity is defined as the involvement (believing, following and/or practicing) of the individual with a religion^10^. Understanding the mechanisms that mediate the association between R/S and health has been a major challenge^10^^,^ 15. The most cited mechanisms include social support, healthy behaviors, religious practices, coping strategies, and cognitive framework^9^^,^ 16. However, these mediators have generated inconsistent results^17^^,^ 18 ^,^ 19.

Sense of coherence is derived from the salutogenic theory, which investigates the factors that allow the maintenance of health, and is divided into three components: 1) Comprehensibility: perception that external or internal stimuli are structured, predictable, and explicable 2) Manageability: perception that the resources to cope with the needs posed by these stimuli are availabl e, and 3) Meaningfulness: perception that these needs are challenges in which it is worth investing and engaging^20^. Studies have shown a positive association between sense of coherence and QoL and inverse associations with indicators of physical and mental illness in several populations^19^^,^ 21.

To test this hypothesis, the objective of the present study was to investigate the association between R/S levels and happiness in patients with CKD who were on hemodialysis and to determine whether this possible association is fully or partially mediated by sense of coherence.

## Methods

### Participants and study setting

A cross-sectional study was conducted involving outpatients from two renal replacement therapy centers in the city of Juiz de Fora, Minas Gerais, Brazil: the University Hospital of the Federal University of Juiz de Fora (HU-UFJF) and NEFROCLIN. Inclusion criteria were adults (≥ 18 years of age), with ≥ 1 year on dialysis, at which point patients are more adapted to therapy. Patients with cognitive deficits that prevented them from answering the questionnaires were excluded.

From the list of patients provided by HU-UFJF, 64 patients were identified; 15 refused to participate, 16 did not meet the inclusion criteria, and 3 were not located, for a total of 30 study participants. Of the 207 patients identified at NEFROCLIN, 14 refused to participate, 35 did not meet the inclusion criteria, 15 had died, 8 had received a transplant, 1 had renal function recovery, and 3 were lost to follow-up, for a total of 131 patients participating in the study. Thus, the final sample contained 161 participants. All participants signed an informed consent form to participate in the study. The study was approved by the Research Ethics Committee of the HU-UFJF, under protocol 375/2011, as well as the Ethics Committees of each treatment center. Data were collected between July 2012 and March 2013.

Self-reporting questionnaires were administered face-to-face by psychology students trained for this study. Throughout the data collection process, regular meetings were held to standardize the study and avoid divergences. Data were collected during hemodialysis sessions at the respective centers, and questionnaires were always administered after the patients had received a snack and were therefore more alert.

### Measures

Socio-demographic data obtained through interviews included age, race/skin color, sex, educational level, marital status, employment status, family income, and religious denomination. Clinical and laboratory data obtained from medical records included hemoglobin, parathyroid hormone (PTHi), KtV (dialysis adequacy index), comorbidities, and time on dialysis. If no data was available for the month in which the survey was applied, data from the month closest to the interview were used.

Religiosity: a validated Portuguese version of the Duke Religious Index (DUREL)^22^^,^ 23 was used. The DUREL has five items, is self-applicable, and indicates the level of religious involvement by addressing three dimensions of religiosity commonly related to health: Organizational Religiosity (OR), Private Religiosity (PR), and Intrinsic Religiosity (IR). All answers are on a Likert scale.

Sense of Coherence: a validated Portuguese version of Antonovsky's Life Orientation Questionnaire/Sense of Coherence Scale^24^^,^ 25 was used. The tool evaluates three dimensions: 1) Comprehensibility, 2) Manageability, and 3) Meaningfulness. Higher scores indicate a stronger sense of coherence with a possible range of 29 to 203.

Happiness: a validated Portuguese version of the Subjective Happiness Scale (SHS)^26^ was used, which is self-applicable and includes 4 items answered on a Likert scale. Higher scores indicate greater happiness, with a possible range of 4 to 28 points.

### Statistical analysis

A descriptive analysis of the data was performed, and data are shown as the mean ± standard deviation or frequency, depending on the characteristics of the variable. We performed a linear regression with happiness as the outcome variable, and adjusted the model for sociodemographic, clinical, and laboratory variables (Model 1). Then, we added each of the religiosity dimensions to the other models, adding Organizational Religiosity to model 2, Private Religiosity to model 3, Intrinsic Religiosity to model 4 and sense of coherence to model 5. Given the association of Private Religiosity, Intrinsic Religiosity, and sense of coherence variables with happiness, two additional models were fitted, the first using Private Religiosity and sense of coherence as predictor variables and the second using Intrinsic Religiosity and sense of coherence, with happiness as the outcome. These models were adjusted for sociodemographic, clinical, and laboratory variables.

The data were analyzed using SPSS (Statistical Package for the Social Sciences) 20.0 for Windows. A 95% confidence interval was adopted.

## Results

The sample consisted predominantly of middle-aged adults (57.8% were between 41 and 64 years old), with low educational level (60.9% had less than a high school education), and unemployed (61.5% retired and 26.7% on medical leave). There was an almost equitable division between men and women and between white, black, and mixed-race individuals, indicating data similar to those of the Brazilian Dialysis Census^27^. The majority of the population was Catholic (59.6%), followed by Protestant (18.6%), and other affiliations (13%). No religious affiliation was reported by 8.7% of the sample (Table 1).

**Table 1:** Demographic characteristics, religiosity, sense of coherence, and happiness (N = 161)

Characteristics		N	*%*
Age group	Up to 40 years	20	12.4
	41 to 64 years	93	57.8
	65 years and over	48	29.8
Race	White	54	33.5
	Black	56	34.8
	Brown	45	28.0
	Other	6	3.7
Gender	Male	87	54.0
Educational level	None	16	9.9
	Up to elementary school	98	60.9
	Up to high school	32	19.9
	College	15	9.3
Marital status	Not married	39	24.2
	Married/Living together	84	52.2
	Widowed/Divorced/Separated	38	23.6
What is your current working situation	Full-Time Employee	1	0.6
	Employed part-time	4	2.5
	Unemployed	2	1.2
	Retired	99	61.5
	Away/Leave or Sickness	43	26.7
	Student	1	0.6
	Others	11	6.8
Income bracket	1 MW or less	102	63.4
	1.1 to 2 MW	59	36.6
Comorbidity	None	29	18.7
	1	79	51
	2	32	20.6
	3	15	9.7
Diabetes	Yes	44	27.3
Religious affiliation	Catholic	96	59.6
	Evangelical	30	18.6
	Others	21	13.0
	None	14	8.7
		Mean	SD
Hb (g/dL)		11.0	1.9
PTHi (pg/mL)		517	413
Kt/V		1.45	0.40
Religiosity	Organizational (1a 6)	4.1	1.7
(min - max)	Private (1 a 6)	4.3	1.7
	Intrinsic (3 a 15)	12.9	2.9
Sense of coherence(min - max)	-	137.2	23.8
Happiness	-	19.7	5.0

SD = Standard deviation; MW = Minimum Wage Hb (g/dl): hemoglobin (g/dL) PTHi: parathyroid hormone (pg/mL) Kt/V: adequacy index in dialysis

In Table 2, we show the positive association between happiness, sense of coherence, Private Religiosity and Intrinsic Religiosity. It is interesting to note that the greater the religiosity, the greater the happiness - the highest coefficients were for Private Religiosity (beta: 0.53) and Intrinsic Religiosity (beta: 0.48). No association was found between organizational religiosity and happiness.

**Table 2:** Linear regression between religiosity and sense of coherence with happiness (adjusted for clinical and laboratory variables)

	Variable	BETA Coefficient	95% Confidence Interval	*p*
Model 1	Organizational religiosity	0.42	-0.11 to 0.95	0.11
Model 2	Private religiosity	**0.53**	0.01 to 1.06	**0.04**
Model 3	Intrinsic religiosity	**0.48**	0.18 to 0.79	**0.002**
Model 4	Sense of coherence	**0.11**	0.09 to 0.15	**< 0.0001**

Adjusted for: age, gender, education, comorbidity, dialysis time (years) and hemoglobin (g/dL), Kt/V (adequacy index in dialysis), and PTH (parathyroid hormone-pg/mL) Statistically significant results (*p* < 0.05) are in bold.

Table 3 shows that, although the sense of coherence had a lower B (beta) coefficient, when evaluated separately with Private Religiosity and IR, it interfered with the coefficients of the variables (see table 2). The betas decreased for Private Religiosity (0.53-0.42) and Intrinsic Religiosity (0.48-0.34), and the association of Private Religiosity and happiness lost significance. The association between Intrinsic Religiosity and happiness remained significant.

**Table 3:** Association between Religiosity and sense of coherence with Happiness (adjusted for sociodemographic and clinical variables)

Variable	BETA Coefficients	Confidence Interval	*p*
Sense of coherence	**0.11**	- 0.09 to 0.15	**< 0.0001**
Private Religiosity	0.42	- 0.03 to 0.88	0.06
Sense of coherence	**0.11**	0.08 to 0.14	**< 0.0001**
Intrinsic Religiosity	**0.34**	0.07 to 0.60	**0.01**

Adjustment was made for age, sex, schooling, comorbidities, dialysis time, hemoglobin (g/dL), Kt/V (dialysis adequacy index), and PTHi (parathyroid hormone. Statistically signifcant results (*p* < 0.05) are in bold.

## Discussion

Our study indicated that R/S is associated with higher levels of happiness and that sense of coherence is an important associated factor of this relationship. The clinical and sociodemographic variables examined were not correlated with happiness.

To our knowledge, this is the first study to investigate levels of happiness and sense of coherence among patients with CKD, as well as to investigate the possible mediating role of sense of coherence and the impact of R/S on health and happiness indicators. Thus, to compare our data with the literature, we used studies with samples that are related (e.g., patients with other chronic diseases) or have related outcomes (e.g., QoL or mental disorders) but not necessarily the same as in the present study.

In sociodemographic terms, our sample is similar to the population of patients on hemodialysis^27^. It is worth noting the high degree of functional/work limitation in the sample, as 88.2% were retired or on work leave, although 70.2% were under 65 years old. The distribution of religious affiliation is close to that of the Brazilian population^28^, which is mostly Catholic, followed by evangelical Christian. The sample had high levels of religious involvement on all three measured dimensions: intrinsic, private, and organizational, corroborating results from other similar samples^11^.

In this study, we found that sense of coherence, Private Religiosity, and Intrinsic Religiosity were consistently correlated with levels of happiness. R/S is generally recognized as a potential coping strategy for addressing the challenges of CKD. Several studies have found that, in patients with CKD, R/S is associated with a decreased perception of the disease negative impact, decreased depression, increased search for social support, and increased QoL and satisfaction^13^^,^ 14 ^,^ 29 ^,^ 30. Evidence from the literature also indicates a usually positive association between levels of religious involvement and levels of positive aspects such as well-being, optimism, and happiness^31^.

Although Organizational Religiosity is usually associated with lower levels of depression and better health, in the present study, it was the only dimension of religiosity that had no association with happiness. A possible explanation is that in situations of greater physical limitation, patients have greater difficulty attending religious meetings and emphasize personal and private religiosity.

An interesting finding was the non-correlation of clinical and laboratory variables with happiness. To our knowledge, this was the first time this association was investigated in patients with CKD. Objective clinical variables often have a much weaker relationship than assumed with subjective assessments of the patient's health status and level of well-being. However, the reasons for this effect are still unclear. Age is associated with worse QoL in studies of patients with CKD^32^^,^ 33. The laboratory variable that correlates most frequently with QoL is the serum hemoglobin level^34^.

Although R/S is usually associated with indicators of health and well-being, the mechanisms that mediate this association is a current knowledge gap^15^^,^ 35.

We hypothesized that sense of coherence would be a possible mediator of the relationship between R/S and happiness. Our findings confirmed our hypothesis, showing a consistent association between R/S and sense of coherence (0.09 to 0.15, *p* < 0.0001). It seems that sense of coherence acts as a full mediator on the impact of Organizational Religiosity (sense of coherence beta: 0.11 (-0.09 to 0.15), *p* < 0.0001 and Private Religiosity beta: 0.42 (0.03 a 0.88), *p* = 0.06) and as a partial mediator on the impact of Intrinsic Religiosity on the levels of happiness in hemodialysis patients (sense of coherence beta: 0.11 (0.08 to 0.14), *p* < 0.0001 and Intrinsic Religiosity beta: 0.34 (0.07 to 0.60), *p* = 0.01).

Other studies have also found a positive association between R/S and sense of coherence^36^^,^ 37. A review showed the effectiveness of religious/spiritual interventions designed to increase participants' sense of coherence^38^.

The present data suggest that R/S and sense of coherence may be targets of psychosocial interventions, such as psychoeducational programs and psychotherapy, that aim to address outcomes in CKD^7^^,^ 39.

Identifying and supporting religious coping strategies and fostering the use of spiritual resources to allow patients to cope better with CKD may be effective strategies.

There is evidence that the simple collection of a patient's spiritual history, even if over a short duration (2-5 minutes), is associated with greater treatment satisfaction and better QoL 40 ^,^ 41. Taking the spiritual history has been recognized as a relatively simple practical way of integrating spirituality into patient care^42^.

The present study has limitations. Because it is a cross-sectional study, causal inferences about the effect of R/S on sense of coherence and happiness should be made with caution. Because our findings are novel, it is important that other researchers attempt to replicate them in longitudinal studies. Care should be taken in generalizing these results to other contexts. This study was conducted in Brazil, where the population is highly religious^28^. However, the clinical and sociodemographic profile of our sample is similar to that of studies conducted in other countries as well as in Brazil, and the impacts of R/S on health have been replicated across a wide range of sociocultural and geographic settings^34^^,^ 43. Another limitation is the absence of data on central nervous system drugs, which was due to difficulty in accessing this information in the medical record.

The strengths of this study include its originality, relatively large sample, use of well-established measures, and broad clinical implications.

## Conclusion

In summary, the present study found for the first time that R/S and sense of coherence are related to the happiness of patients on hemodialysis. No sociodemographic, clinical, or laboratory variables were associated with patients' levels of happiness. In view of the increasing value and attention given to the well-being and QoL of patients with CKD, R/S should be better investigated as possible components of psychosocial interventions that, together with drug and renal replacement therapies, may promote better QoL.
